# Medical and Household Characteristics Associated with Methicillin Resistant *Staphylococcus aureus* Nasal Carriage among Patients Admitted to a Rural Tertiary Care Hospital

**DOI:** 10.1371/journal.pone.0073595

**Published:** 2013-08-26

**Authors:** Leah Schinasi, Steve Wing, Pia D. M. MacDonald, David B. Richardson, Jill R. Stewart, Kerri L.Augustino, Delores L. Nobles, Keith M. Ramsey

**Affiliations:** 1 Department of Epidemiology, The University of North Carolina at Chapel Hill, Chapel Hill, North Carolina, United States of America; 2 Social & Scientific Systems, Inc., Durham, North Carolina, United States of America; 3 Department of Environmental Sciences and Engineering, The University of NC at Chapel Hill, Chapel Hill, North Carolina, United States of America; 4 Department of Infection Control, Vidant Medical Center, Greenville, North Carolina, United States of America; 5 Division of Infectious Diseases, The Brody School of Medicine at East Carolina University, Greenville, North Carolina, United States of America; University of Iowa, United States of America

## Abstract

**Background:**

Methicillin resistant *Staphylococcus aureus* (MRSA) poses a threat to patient safety and public health. Understanding how MRSA is acquired is important for prevention efforts. This study investigates risk factors for MRSA nasal carriage among patients at an eastern North Carolina hospital in 2011.

**Methods:**

Using a case-control design, hospitalized patients ages 18 – 65 years were enrolled between July 25, 2011 and December 15, 2011 at Vidant Medical Center, a tertiary care hospital that screens all admitted patients for nasal MRSA carriage. Cases, defined as MRSA nasal carriers, were age and gender matched to controls, non-MRSA carriers. In-hospital interviews were conducted, and medical records were reviewed to obtain information on medical and household exposures. Multivariable conditional logistic regression was used to derive odds ratio (OR) estimates of association between MRSA carriage and medical and household exposures.

**Results:**

In total, 117 cases and 119 controls were recruited to participate. Risk factors for MRSA carriage included having household members who took antibiotics or were hospitalized (OR: 3.27; 95% Confidence Interval (CI): 1.24–8.57) and prior hospitalization with a positive MRSA screen (OR: 3.21; 95% CI: 1.12–9.23). A lower proportion of cases than controls were previously hospitalized without a past positive MRSA screen (OR: 0.40; 95% CI: 0.19–0.87).

**Conclusion:**

These findings suggest that household exposures are important determinants of MRSA nasal carriage in hospitalized patients screened at admission.

## Introduction

When it was first identified in the 1960s, Methicillin resistant *Staphylococcus aureus* (MRSA) was a nosocomial pathogen [1]. In the 1990s, the epidemiology of this virulent pathogen changed; it was identified in people without recent medical contact [2]. Healthcare associated (HA) and community associated (CA) MRSA strains are genetically distinct [3]. However, CA strains have been detected in hospital outbreaks [4], and people without recent hospitalizations may carry HA strains [5]. Indeed, the epidemiology of MRSA is complex and distinctions between CA and HA strains are increasingly difficult to disentangle.

In addition to medical related exposures [6], household and environmental exposures are gaining recognition as potentially important determinants of transmission and acquisition [7], [8]. For example, MRSA carriage or infection has been associated with living with household members with a history of infections [8], [9] and with presence of MRSA in the household environment [8]. There is evidence that MRSA may be transmitted between humans and animals [9], [10]. There have been reports of MRSA infections and outbreaks among athletics participants [11] and associations between smoking and MRSA infection [12]. Genotypic, clinical and demographic differences in the epidemiology of MRSA have been observed among regions and sub-communities within the same area [13], [14]. Therefore, region specific investigations of the sources of acquisition are important for informing infection prevention.

Although there is a growing body of literature on risk factors for MRSA carriage in urban communities [8], [15], [16], data on predictors of carriage in rural areas remains limited. The eastern section of North Carolina is largely rural [17], and compared to the statewide population, a higher proportion of eastern North Carolina residents are Black and live in poverty–approximately 28% and 20% of eastern North Carolinians, respectively, versus 21% and 17% of North Carolina’s total population [18]. Vidant Medical Center (VMC) is an 861-bed teaching hospital of the Brody School of Medicine at East Carolina University and the tertiary care center for 29 eastern North Carolina counties. Since February of 2007, VMC has screened all patients for MRSA within 24 hours of admission using duplicate swabs of the anterior nares. Since the initiation of this program, all MRSA carriers have been placed on contact isolation, prescribed 5-day courses of mupirocin for application to their nares, and bathed with chlorhexidine soap [19].

VMC’s screening program provided the opportunity to investigate risk factors for MRSA carriage among hospitalized patients in eastern North Carolina. The objective of this study was to investigate household exposures, hospitalization and/or MRSA screening history, demographic characteristics, and smoking history as predictors of MRSA nasal carriage in patients who were recently admitted and screened at VMC. To investigate differences in the epidemiology of the genetic subtypes of MRSA, a repetitive sequence-based polymerase chain reaction (rep-PCR) [20] was used to characterize isolates from study participants as CA or HA, and associations between exposures and CA or HA MRSA carriage were investigated.

## Materials and Methods

### Ethics statement

The Institutional Review Boards at the University of North Carolina at Chapel Hill and East Carolina University approved this work. Participants provided written informed consent and signed Health Information Portability and Accountability Act authorization forms.

### Participant eligibility

Eligible participants were admitted patients at VMC, screened for MRSA within 24 hours of their admission and English or Spanish speakers. The same eligibility criteria were applied to cases and controls. These data arise from a parent study, the objectives of which were to investigate relationships between occupational exposures to livestock and MRSA nasal carriage. To increase the probability of employment, participants were restricted to ages 18–65. To increase the probability of exposure to livestock for the parent study, participants were restricted to residents of swine-producing zip codes in which the permitted number of swine was equal to or greater than the median number listed with the North Carolina Division of Water Quality (North Carolina zip codes with 1,032,750 or more permitted swine). Of 1,083 zip codes in North Carolina [21], 176 were eligible.

### Case identification

Cases were defined as inpatients who screened positive for MRSA nasal carriage upon admission to the hospital. They were identified by reviewing daily reports from the electronic medical record, which captured MRSA screening results and demographic data on all admitted patients to the hospital.

### Control identification

Controls were defined as inpatients who screened negative for MRSA upon admission to the hospital. For every case, an eligible control was identified by reviewing the same daily reports of the electronic medical record that were used to identify cases. When more than one patient was eligible to serve as a control, a random number generator was used to select the control. If that potential control declined to participate or was discharged from the hospital before the interviewer approached them, another control was identified. Because of the above-stated objective of the parent study to investigate the relationship of MRSA carriage with occupational exposures, and because gender and age are strong potential determinants of occupational exposure and case status that could be controlled by design rather than analysis only, controls were matched to cases based on age (±5 years) and gender.

### Interviews and medical record review

Participant enrollment and structured interviews were conducted with cases and controls from July – December 2011. The interviewer approached eligible cases and controls in their hospital rooms, introduced the study, and invited them to participate in a brief interview. The structured interview included questions about medical and antibiotic use history in the past 12 months for participants and their household member(s), indoor pets, sports participation or gym use in the past 2 weeks, smoking history in the past 12 months, and demographics. One author (L.S.) conducted all interviews.

The following information was abstracted from medical records: prior MRSA screening results, surgery, hospitalizations, and antibiotic prescriptions within the past 12 months; and diagnoses listed in the discharge summary for the current hospitalization. If participants reported not taking antibiotics but their medical records showed antibiotic prescriptions, including mupirocin for previous MRSA carriage, the data were coded to reflect the record. If participants reported antibiotic use but their medical records showed no antibiotic prescriptions, participants’ reports were used. Because of the way the question about antibiotic use was asked during the interview, it was not possible to tell if participants self-reported mupirocin use as an antibiotic exposure; therefore, mupirocin prescriptions recorded in the medical chart were included with other antibiotic exposures. One author (L.S) abstracted all medical record data.

### MRSA detection and typing

VMC’s clinical microbiology laboratory used the BD GenOhm® MRSA polymerase chain reaction (PCR) to test nasal swabs for MRSA [22]. The BD GeneOhm® real-time PCR identifies MRSA by targeting the junction of the *SCCmec* right extremity (SRE) with a section of the *orf*X gene, which is specific to *S. aureus* [23]. Swabs were stored at 4°C for 24 – 48 hours, then transferred to the infection control laboratory and streaked onto a CHROMagar® MRSA plate (CHROM agar Microbiology, Paris, France). The CHROMagar® plate was incubated for 24 – 48 hours at 37°C. Mauve colonies were identified as MRSA.

DNA was extracted using an UltraClean™ Microbial DNA Isolation Kit (Mo Bio Laboratories, Solana Beach, CA). The NanoDrop® ND-1000 Spectrophotometer (Isogen, Ijssel stein, The Netherlands) was used to estimate the genomic DNA concentrations. Extracts were diluted to give a final DNA concentration of 35 ng/µl.

The DiversiLab® Staphylococcus kit for DNA fingerprinting (bioMerieux, Boxtel, Netherlands), a repetitive sequence-based PCR (rep-PCR), was used to amplify regions between repetitive, noncoding sequences in DNA samples. The Diversilab® system is a commercially available strain typing kit that is popular in clinical settings because it is less time consuming compared to pulsed-field gel electrophoresis and, unlike sequence based typing methods such as *Staphylococcus* Protein A (spa) or multi locus sequence typing, does not require access to DNA sequencing facilities [20]. The protocol was run according to manufacturer specifications. Software from the DiversiLab® system (version v.r.3.3.40) was used for genotyping [20]. DiversiLab® produces a dendogram, a graph of fluorescence intensity that corresponds to banding patterns, and a similarity matrix. The rep-PCR profiles were compared to the DiversiLab® MRSA library, which contains 70 samples of 14 representative USA pulsed field gel electrophoresis types [3]. Fingerprints with no banding differences were considered matches and assigned the library type. Isolates without a match in the library were considered non-typeable, or no match. MRSA isolates were subsequently classified as CA, HA or non-matches.

### Statistical analysis

Conditional logistic regression models, which adjusted for matching by age and gender, were used to estimate odds ratios (OR) and 95% confidence intervals (CIs). The following variables were examined in a multivariable model: education; self-reported race/ethnicity; visiting a gym or playing sports; tobacco smoking history; indoor cats or dogs; household member presence, hospitalization or antibiotic use; and participant hospitalization and MRSA screening history. These variables were selected *a priori*, based on the hypothesis that they might be associated with MRSA nasal carriage. Variables were coded to provide the best predication with the fewest degrees of freedom, as indicated by Akaike Information Criterion (AIC) statistics. The variable representing the presence, hospitalization and antibiotic use history of household members was coded as a 3-level categorical variable (0 =  participant lived alone, 1 =  participant lived with 1 or more household members, but none used antibiotics in the past 4 weeks or were hospitalized in the past 12 months, 2 = participant lived with 1 or more household members who used antibiotics in the past 4 weeks or were hospitalized in the past 12 months). Similarly, history of participant hospitalization and MRSA screening was coded as a 3-level categorical variable (0 = not hospitalized in the past 12 months, 1 = hospitalized but never screened positive for MRSA in the past 12 months, 2 = hospitalized and screened positive for MRSA at least once in the past 12 months). These variables were coded to create mutually exclusive categories that could be included in the same multivariable model. All the other variables included in the multivariable model were coded dichotomously.

In addition to the analysis of the full data set, cases whose isolates grew MRSA colonies were compared to their matched controls. Also, the relationship between HA or CA MRSA carriage and the explanatory variables was examined. In the analysis of CA and HA carriers, samples sizes were small; therefore, each explanatory variable was investigated in a separate model.

Because patients were not always available to be interviewed due to rapid turnover or medical treatments and surgery, and because not all eligible patients agreed to participate, at the end of data collection some participants were left without a matching case/control. To avoid double loss of information, in analysis, case and control matched sets were pooled. Pooled sets were created by identifying the appropriate gender/age match who was admitted within the shortest amount of time of the unmatched case/control. The use of the same control for more than one case has been described previously as a valid method that should not bias measures of association [24].

All statistical analyses were performed using Statistical Analysis Software version 9.3.

## Results

Of 164 cases and 190 controls invited, 121 (73.8%) and 122 (64.2%) consented to participate, respectively. Subsequently, 4 cases and 3 controls were excluded based on having addresses outside the eligible zip codes, leaving 117 cases and 119 controls for analysis. One hundred (89.3%) matched sets had 1 case per control. Because cases and controls were pooled to avoid loss of information, 7 sets (6.3%) had 2 controls per case and 5 (4.5%) had 2 cases per control. All participants lived in 22 counties in eastern North Carolina or in the eastern-most portion of central NC.

Sixty-eight (57.1%) controls and 67 (57.3%) cases were female (Table 1). Fifty-eight controls (48.7%) and 54 (46.2%) cases identified as non-Hispanic white. In the past 12 months, 94 cases (80.3%) and 93 controls (78.2%) used or were prescribed antibiotics. Cases and controls had similar numbers of household members.

**Table 1:** Characteristics of methicillin resistant *Staphylococcus aureus* nasal carriers and their gender and age matched controls.

	No. (%)			
	Controls		Cases	
	(n = 119)		(n = 117)	
Female	68	57.1	67	57.3
Age, y				
18–29	29	24.4	24	20.5
30–39	15	12.6	16	13.7
40–49	17	14.3	22	18.8
50–59	40	33.6	37	31.6
60–65	18	15.1	18	15.4
Race or ethnicity^a^				
Non-Hispanic White	58	48.7	54	46.2
Non-Hispanic Black	55	46.2	53	45.3
Hispanic or Latino	3	2.5	4	3.4
Other	3	2.5	6	5.1
No. household members				
Mean and standard error	3.2	5.5	3.1	1.9
Min and max	0	>10	0	>10
Antibiotic use, past 12 mo.^b^	93	78.2	94	80.3
Surgery, past 12 mo.	40	33.6	46	39.3
Hospitalized, past 12 mo.	70	58.8	71	60.7

Abbreviations: y, year; mo, month; min, minimum; max, maximum.

a Race/ethnicity was self-reported by participants during the interview.

The other category includes participants identifying their race or ethnicity

as Asian, American Indian, or other.

b Any participant who was previously prescribed mupirocin for MRSA

nasal carriage within the past year was coded as having taken antimicrobials.

Five cases had concomitant MRSA clinical infections—abscesses (n = 2), cellulitis/abscess, pneumonia, and bacteremia (Table S1). Thirteen (11.1%) cases and 3 (3.4%) controls had cellulitis or soft tissue infection diagnoses. Seven (6.0%) cases and 3 (2.5%) controls had diarrhea. Thirty-eight (31.9%) cases and 31 (26.5%) controls had diabetes.

Odds ratios were derived from a conditional, multivariable model (Table 2). Cases had a higher odds of having less than a high school degree (OR: 2.04, 95% CI: 0.75–5.50), being a non-white race, having indoor cats or dogs (OR: 1.79; 95% CI: 0.88–3.63), smoking cigarettes in the past 12 months (OR: 1.60; 95% CI: 0.80–3.19), and visiting a gym or playing sports in the past 2 weeks (OR: 2.64; 95% CI: 0.64–10.88). Cases had a higher odds of past hospitalization with a positive MRSA screen (OR: 3.21; 95% CI: 1.12–9.23) but a lower odds of hospitalization without a positive screen in the past 12 months (OR: 0.40; 95% CI: 0.19–0.87). Additionally, cases had 3.27 times the odds of living with someone who had used antibiotics in the past 4 weeks and/or was hospitalized in the past 12 months (95% CI: 1.24–8.57).

**Table 2:** Estimates of association of methicillin resistant *Staphylococcus aureus* (MRSA) nasal carriage identified by polymerase chain reaction with medical and household exposures from a multivariable logistic model conditioned on age and gender.

	No. ( %)					
	Controls		Cases		OR	95% CI
	(n = 119)		(n = 117)			
At least high school or general education development degree	106	89.1	93	79.5	1.00	-
Less than high school or general education development degree	13	10.9	24	20.5	2.04	0.75–5.50
No cats or dogs inside the home	79	66.4	72	61.5	1.00	-
Cats or dogs inside the home	40	33.6	45	38.5	1.79	0.88–3.63
Non-Hispanic white race or ethnicity^a^	58	48.7	54	46.2	1.00	-
Hispanic and/or non-white race or ethnicity	61	51.3	63	53.9	1.48	0.70–3.11
Did not smoke tobacco cigarettes in the past 12 mo.	76	63.9	65	55.6	1.00	-
Smoked tobacco cigarettes in the past 12 mo.	43	36.1	52	44.4	1.60	0.80–3.19
Did not visit a gym or participate in sports in the past 2 weeks^b^	114	95.8	108	92.3	1.00	-
Visited a gym or participated in sports in the past 2 weeks	5	4.2	9	7.7	2.64	0.64–10.88
Prior hospitalization and MRSA nasal carriage in the past 12 mo.^c^						
Not hospitalized in the past 12 mo.	49	41.2	46	39.3	1.00	-
Hospitalized and never screened positive for MRSA in the past 12 mo.	64	53.8	41	35.0	0.40	0.19–0.87
Hospitalized and screened positive for MRSA at least once in the past 12 mo.	6	5.0	30	25.6	3.21	1.12–9.23
Household members c						
No household members	23	19.3	15	12.8	1.00	-
Household members did not use antibiotics in the past 4 weeks and not hospitalized in the past 12 mo.	71	59.7	58	49.6	1.42	0.59–3.45
Household members used antibiotics in the past 4 weeks and/or hospitalized in the past 12 mo.	25	21.0	44	37.6	3.27	1.24–8.57

Abbreviation: months, mo.; odds ratio, OR; confidence interval, CI.

a Non-white or Hispanic includes non-Hispanic black, Hispanic/Latino, Asian, American Indian, or other race/ethnicities.

b The gym visitation/sports participation variable reflects the 2 weeks prior to the hospital admission, even though 2 cases and 1 control were screened for MRSA 9 or more days before their current hospital admission.

c Entered into the model as a 3-level categorical variable.

Of 117 cases, 108 duplicate swabs were available for culturing and 49 (45.4%) grew MRSA colonies. Using the multivariable model, the 49 culture positive cases were compared to 52 matched controls (Table S2). Because of the small numbers of participants in these analyses, the resulting measures of association were imprecise. Being hospitalized but never screening positive for MRSA was negatively associated with MRSA carriage (OR: 0.29; 95% CI: 0.06–1.39). Living with someone who used antibiotics in the past 4 weeks and/or was hospitalized in the past year (OR: 4.76; 95% CI: 0.88–25.72), having less than a high school degree (OR: 2.65, 95% CI: 0.53–13.24); and having indoor pets (OR: 1.26; 95% CI: 0.40–3.99) was associated with a higher odds of MRSA carriage. Similar proportions of cultured cases and controls had prior hospitalization in addition to prior MRSA carriage (OR: 1.03; 95% CI: 0.25–4.24). Also, lower proportions of culture positive cases than controls were non-Hispanic white (OR: 0.89; 95% CI: 0.26–3.09), smoked cigarettes in the past year (OR: 0.61; 95% CI: 0.14–2.62), and lived with someone who did not use antibiotics and was not hospitalized (OR: 0.60; 95% CI: 0.15–2.46). Because of sparse data, the effect of gym use or sports participation in the past 2 weeks was not estimable.

### Molecular typing

Of 49 isolates typed via rep-PCR, 7 (14.3%) did not match types in the DiversiLab® library. Twenty (40.8%) isolates matched the CA strain, USA300. The other 22 isolates matched HA strains; 15 (30.6%) were USA100, 2 (4.1%) were USA500, 3 (6.1%) were USA800, 1 (2.0%) was USA200, and 1 (2.0%) was USA600. None of the non-typeable strains had a pattern that matched the livestock associated multi locus sequence type 398 strain in the DiversiLab® typing system [25].

CA and HA carriers were compared with controls using conditional logistic regression models (Table S3). Because there were only 20 CA isolates and 22 HA isolates in these analyses, the resulting OR estimates were imprecise. However, a higher proportion of CA carriers but a lower proportion of HA carriers were previously hospitalized with a past positive MRSA screen (OR: 7.67; 95% CI: 0.86–68.38; OR: 0.54; 95% CI: 0.10–2.90); had indoor pets (OR: 1.93; 95% CI: 0.49–7.58; OR: 0.60; 95% CI: 0.14–2.51); were a non-white race (OR: 1.49; 95% CI: 0.32–6.91; OR: 0.60; 95% CI: 0.14–2.51); and had household members who did not use antibiotics in the past 4 weeks and were not hospitalized in the past year (OR: 1.19; 95% CI: 0.19–7.53 and OR: 0.89; 95% CI:0.18–4.48). Compared to controls, higher proportions of CA and HA carriers had less than a high school degree (OR: 2.56; 95% CI: 0.46–14.29; OR: 1.50; 95% CI: 0.25–8.98) and smoked cigarettes in the past year (OR: 2.36; 95% CI: 0.44–12.56; OR: 1.67; 95% CI: 0.40–6.97). Higher proportions of CA carriers had household members who used antibiotics in the past 4 weeks and/or were hospitalized in the past 12 months (OR: 3.64; 95% CI: 0.34–39.22). Due to sparse data, this effect was not estimable for HA carriers, and the effect of gym use/sports participation was not estimable for CA or HA carriers.

## Discussion

From the inception of the universal MRSA screening program at VMC in February of 2007 until December of 2011, approximately 7.9% of nearly 225,000 screened patients, excluding the neonatal population, were identified as MRSA nasal carriers by PCR. We compared participants who were hospitalized and screened positive for MRSA on a previous occasion within the past year to participants who were not hospitalized in the past year; MRSA carriage identified by PCR predicted nasal carriage at later hospital admission. VMC prescribes mupirocin to MRSA carriers to decolonize their nares; patients complete the treatment either while they are hospitalized or as outpatients. Of 36 participants who screened positive for MRSA carriage on a previous visit to VMC within the past year, all but 1 were prescribed mupirocin after their last positive MRSA screen. This suggests that MRSA carriers who retested positive were re-colonized in the community.

The finding that past MRSA carriage predicted a positive MRSA screen upon readmission could be partially explained by carriage of mupirocin resistant MRSA strains. Based on point prevalence estimates, low- and high-level mupirocin resistance was detected in 3.5% and 6.0% of isolates collected from VMC patients in 2011, respectively (unpublished data). Therefore, it is unlikely that mupirocin resistance fully explains these results.

Participants who were hospitalized in the past 12 months without a prior positive MRSA screen had lower odds of MRSA carriage than participants who were not hospitalized in the past year. Previous research suggests that past hospitalization is associated with MRSA carriage [15], [26], [27]. Patients previously hospitalized with a negative MRSA PCR on admission may be less susceptible or less exposed in the community.

Patterns of MRSA carriage are commonly classified as persistent, intermittent, and absent. Previous research has shown that a high percentage of patients who initially screen negative for MRSA return to the hospital with negative screens on future visits, whereas high proportions of those who screen positive on an initial visit show patterns of persistent or intermittent carriage [28]. Although results should be interpreted tentatively since most of the measures of association were imprecise due to sparse data, results from our study contribute to evidence that the household environment may affect MRSA acquisition [7], [8], [29], [30] and could play a role in intermittent or persistent carriage. Compared to living alone, living with household members who either used antibiotics or were hospitalized was positively associated with MRSA carriage. It is possible that household members acquired MRSA through past medical related exposures and then exposed MRSA carriers in this study.

Of 49 culture positive isolates, 22 (44.9%) matched HA types, 20 (40.8%) matched CA types and 7 (14.3%) did not have a match in the DiversiLab® MRSA library. To explore potential differences in the epidemiology of HA versus CA MRSA, we examined associations between carriage of each MRSA type and the exposure variables. Because of small numbers of participants in these analyses, the measures of association were imprecise. However, a higher proportion of CA carriers than controls were hospitalized and screened positive for MRSA previously. Other studies have reported that CA strains may be carried by those with a history of hospitalization [5]. Based on small numbers, the proportion of culture positive cases carrying CA strains was high compared to that reported by others [5], [15]. The high proportion of cases carrying CA MRSA in our study could be attributable to the exclusion of patients over age 65, other unique characteristics of the study population, or the relatively low yield (45%) of cultures grown and typed from MRSA nasal carriers identified by PCR.

This study was non-randomized. In recent decades, emphasis on p-values and statistical tests of significance has been strongly discouraged in non-randomized study for several reasons [31]. Therefore, we have interpreted ORs based on their magnitude and biological plausibility, using 95% CIs as measures of precision rather than tests of significance.

These results must be interpreted cautiously. The BD GenOhm® real-time PCR identified the 117 cases. After subculturing on selective media, MRSA was recovered from 49 of 108 available duplicate swabs of the anterior nares. The PCR methodology has been shown to be more sensitive than selective media, which might account for some of the higher detection [22]. However, these data could also raise concerns about false positives among the cases identified by PCR, which might have detected methicillin sensitive *S. aureus* containing SCC*mec* fragments that lack the mecA gene, non-viable bacteria, or DNA residue that remained following decolonization [23], [32], [33], [34]. The percentage of PCR positive swabs that did not grow colonies was similar to that reported in some but not all studies. For example, in a neonatal intensive care unit, MRSA was detected by PCR in 21 of 435 (4.8%) nasal swabs, and 11 (52.4%) of the PCR positive swabs grew MRSA colonies when cultured [35]. In another study, 123 of 599 nasal swabs were positive by PCR, of which 77 (62.6%) were positive by culture without enrichment [36]. In a third study, 606 nose, throat, and groin/perineum swabs were collected from 202 patients; 120 MRSA specimens were detected by PCR, of which 85 (70.8%) were detected by direct culture [33]. In our study, the delay from the time when the duplicate swabs from PCR positives were transported and plated onto selective media varied from 24 – 48 hours, which could have reduced subsequent culture yields. Additionally, a higher proportion of cases might have grown colonies on selective media had overnight enrichment been used, which has been shown to improve the sensitivity of cultures [37].

The 236 participants of this study were residents of 22 North Carolina counties. Based on United States Census data, percentages of Black residents in these counties ranged from 15% to 62% [18]. By comparison, 21% of all North Carolina residents are Black [18]. Therefore, results from this study might not be generalizable to members of other North Carolina communities, or to non-hospitalized populations.

Because VMC only screens patients’ anterior nares, people with MRSA on other regions of the body could have been misidentified as controls. Additionally, although cases could have been carrying multiple MRSA strains [38], DNA of only 1 bacterial colony per culture-positive swabs was extracted and typed. Finally, participants’ knowledge of their screening results could have influenced their interview responses.

This study has several strengths. One author (L.S.) conducted all interviews and abstracted data from medical records, which helped maintain internal consistency. By conducting in-hospital interviews, we collected information that would be unavailable by record review alone. By using information in patients’ medical records, the quality of the interview data was improved. Overall, there were small amounts of missing data and participation rates were high. Using the rapid PCR testing methodology to screen patients for MRSA allowed the interviewer to identify and approach eligible patients soon after they were admitted [22].

Community and household exposures may influence MRSA carriage and VMC’s hospital-based screening program may be detecting carriage among those exposed to MRSA. Further study of the relationship between MRSA carriage and community exposures, and continued surveillance for strains entering hospitals will help elucidate the dynamic epidemiology of MRSA.

## Supporting Information

Table S1 **Distribution of selected comorbidities, diagnoses, and symptoms listed in the medical record discharge summaries of methicillin resistant** ***Staphylococcus aureus*** **(MRSA) nasal carriers and controls.**
(DOCX)

Table S2 **Estimates of association of methicillin resistant** ***Staphylococcus aureus*** **(MRSA) nasal carriage identified by culture with medical and household exposures from a multivariable logistic model conditioned on age and gender.**
(DOCX)

Table S3 **Estimates of association of health care and community associated methicillin resistant** ***Staphylococcus aureus*** **(MRSA) nasal carriage with medical and household exposures from conditional logistic regression models.**
(DOCX)
