# Supplementary material for: Medical and Household Characteristics Associated with Methicillin Resistant Staphylococcus aureus Nasal Carriage among Patients Admitted to a Rural Tertiary Care Hospital
Source: PLoS One. 2013 Aug 26;8(8):e73595. doi: 10.1371/journal.pone.0073595 (PMC3753306; doi:10.1371/journal.pone.0073595)
Supplement: Table S1 — Distribution of selected comorbidities, diagnoses, and symptoms listed in the medical record discharge summaries of methicillin resistant Staphylococcus aureus (MRSA) nasal carriers and controls. (DOCX) [file pone.0073595.s001.docx]

| **Table S1.** Distribution of selected comorbidities, diagnoses, and symptoms listed in the medical record discharge summaries of methicillin resistant *Staphylococcus aureus* (MRSA) nasal carriers and controls^a^ | | | | |
| --- | --- | --- | --- | --- |
|  | **No. ( %)** | | | |
|  | **Controls** | | **Cases** | |
|  | **(n=119)** | | **(n=117)** | |
| End-stage renal disease | 5 | 4.2 | 7 | 6.0 |
| Cancer | 7 | 5.9 | 5 | 4.3 |
| Diabetes mellitus | 38 | 31.9 | 31 | 26.5 |
| HIV or AIDS | 2 | 1.7 | 4 | 3.4 |
| MRSA infection | 0 | - | 5 | 4.3 |
| Sepsis or bacteremia | 1 | 0.8 | 5 | 4.3 |
| Pneumonia | 2 | 1.7 | 1 | 0.9 |
| Urinary tract infection | 4 | 3.4 | 2 | 1.7 |
| Necrotizing fasciitis | 0 | - | 1 | 0.9 |
| Cellulitis or soft tissue infection | 3 | 3.4 | 13 | 11.1 |
| Abscess | 8 | 6.7 | 8 | 6.8 |
| Fever | 3 | 2.5 | 3 | 2.6 |
| Diarrhea | 3 | 2.5 | 7 | 6.0 |
| Nausea or vomiting | 7 | 5.9 | 7 | 6.0 |
| Shortness of breath | 3 | 2.5 | 4 | 3.4 |
| Chest pain | 7 | 5.9 | 6 | 5.9 |
| Abbreviations: human immunodeficiency virus, HIV; acquired immune deficiency syndrome, AIDS  ^a^The categories listed are not mutually exclusive. | | | | |
