# Supplementary material for: Medical and Household Characteristics Associated with Methicillin Resistant Staphylococcus aureus Nasal Carriage among Patients Admitted to a Rural Tertiary Care Hospital
Source: PLoS One. 2013 Aug 26;8(8):e73595. doi: 10.1371/journal.pone.0073595 (PMC3753306; doi:10.1371/journal.pone.0073595)
Supplement: Table S2 — Estimates of association of methicillin resistant Staphylococcus aureus (MRSA) nasal carriage identified by culture with medical and household exposures from a multivariable logistic model conditioned on age and gender. (DOCX) [file pone.0073595.s002.docx]

**Table S2.** Estimates of association of methicillin resistant *Staphylococcus aureus* (MRSA) nasal carriage identified by culture with medical and household exposures from a multivariable logistic model conditioned on age and gender

|  |  | **No (%)** | | |  |  |  |
| --- | --- | --- | --- | --- | --- | --- | --- |
|  | **Controls** | | **Cases** | | | **OR** | **95% CI** |
|  | **(n=52)** | | **(n=49)** | | |  |  |
| At least high school or general education development degree | 47 | 90.4 | 40 | 81.6 | | 1.00 | - |
| Less than high school or general education development degree | 5 | 9.6 | 9 | 18.4 | | 2.65 | 0.53-13.25 |
| No cats or dogs inside the home | 34 | 65.4 | 33 | 67.4 | | 1.00 | - |
| Cats or dogs inside the home | 18 | 34.6 | 16 | 32.7 | | 1.26 | 0.40-3.99 |
| Non-Hispanic white race/ethnicity^a^ | 23 | 44.2 | 23 | 46.9 | | 1.00 | - |
| Hispanic and/or non-white race/ethnicity^a^ | 29 | 55.8 | 26 | 53.1 | | 0.89 | 0.26-3.09 |
| Did not smoke tobacco cigarettes in the past 12 mo. | 36 | 69.2 | 31 | 63.3 | | 1.00 | - |
| Smoked tobacco cigarettes in the past 12 mo. | 16 | 30.8 | 18 | 36.7 | | 0.61 | 0.14-2.62 |
| Did not visit a gym or participate in sports in the past 2 weeks^b^ | 51 | 98.1 | 46 | 93.9 | | 1.00 | - |
| Visited a gym or participated in sports in the past 2 weeks^b^ | 1 | 1.9 | 3 | 6.1 | | NE | NE |
| Prior hospitalization and MRSA nasal carriage in past 12 mo.^c^ |  |  |  |  | |  |  |
| Not hospitalized in the past 12 mo. | 25 | 48.1 | 23 | 46.9 | | 1.00 | - |
| Hospitalized and never screened positive for MRSA in the past 12 mo. | 21 | 40.4 | 14 | 28.6 | | 0.29 | 0.06-1.39 |
| Hospitalized and screened positive for MRSA at least once in the past 12 mo. | 6 | 11.5 | 12 | 24.5 | | 1.03 | 0.25-4.24 |
| Household members^c^ |  |  |  |  | |  |  |
| No household members | 8 | 15.4 | 8 | 16.3 | | 1.00 | - |
| Household members did not use antibiotics in the past 4 weeks and not hospitalized in the past 12 mo. | 37 | 71.2 | 20 | 40.8 | | 0.60 | 0.15-2.46 |
| Household members used antibiotics in the past 4 weeks and/or was hospitalized in the past 12 mo. | 7 | 13.5 | 21 | 42.9 | | 4.76 | 0.88-25.72 |

Abbreviation: months, mo.; odds ratio, OR; confidence interval, CI; non-estimable effect estimate, NE

^a^ Non-white or Hispanic includes non-Hispanic black, Hispanic/Latino, Asian, American Indian, or other race/ethnicities.

^b^The gym visitation/sports participation variable reflects the 2 weeks prior to the hospital admission.

^c^ Entered into the model as a 3-level categorical variable.
