# Supplementary material for: Medical and Household Characteristics Associated with Methicillin Resistant Staphylococcus aureus Nasal Carriage among Patients Admitted to a Rural Tertiary Care Hospital
Source: PLoS One. 2013 Aug 26;8(8):e73595. doi: 10.1371/journal.pone.0073595 (PMC3753306; doi:10.1371/journal.pone.0073595)
Supplement: Table S3 — Estimates of association of health care and community associated methicillin resistant Staphylococcus aureus (MRSA) nasal carriage with medical and household exposures from conditional logistic regression models. (DOCX) [file pone.0073595.s003.docx]

|  | **Health care associated MRSA** | | | | | | **Community associated MRSA** | | | | | |
| --- | --- | --- | --- | --- | --- | --- | --- | --- | --- | --- | --- | --- |
|  | **No. (%)** | | | |  | | **No. (%)** | | | |  | |
|  | **Controls** | | **Cases** | | **OR** | **95% CI** | **Controls** | | **Cases** | | **OR** | **95% CI** |
|  | **(n=23)** | | **(n=22)** | |  |  | **(n=23)** | | **(n=20)** | |  |  |
| At least high school or general education development degree | 21 | 91.3 | 19 | 86.4 | 1.00 | - | 20 | 87.0 | 15 | 75.0 | 1.00 | - |
| Less than high school or general education development degree | 2 | 8.7 | 3 | 13.6 | 1.50 | 0.25-8.98 | 3 | 13.0 | 5 | 25.0 | 2.56 | 0.46-14.29 |
| No cats or dogs inside the home | 16 | 69.6 | 17 | 77.3 | 1.00 | - | 15 | 65.2 | 11 | 55.0 | 1.00 | - |
| Cats or dogs inside the home | 7 | 30.4 | 5 | 22.7 | 0.60 | 0.14-2.51 | 8 | 34.8 | 9 | 45.0 | 1.93 | 0.49-7.58 |
| Non-Hispanic white race/ethnicity^a^ | 8 | 34.8 | 10 | 45.5 | 1.00 | - | 13 | 56.5 | 10 | 50.0 | 1.00 | - |
| Hispanic and/or non-white race/ethnicity | 15 | 65.2 | 12 | 54.6 | 0.60 | 0.14-2.51 | 10 | 43.5 | 10 | 50.0 | 1.49 | 0.32-6.91 |
| Did not smoke tobacco cigarettes in the past 12 mo. | 18 | 78.3 | 15 | 68.2 | 1.00 | - | 15 | 65.2 | 11 | 55.0 | 1.00 | - |
| Smoked tobacco cigarettes in the past 12 mo. | 5 | 21.7 | 7 | 31.8 | 1.67 | 0.40-6.97 | 8 | 34.8 | 9 | 45.0 | 2.36 | 0.44-12.56 |
| Did not visit a gym or participate in sports in the past 2 weeks | 22 | 95.7 | 21 | 95.5 | 1.00 | - | 22 | 100 | 18 | 90.0 | 1.00 | - |
| Visited a gym or participated in sports in the past 2 weeks | 1 | 4.4 | 1 | 4.6 | NE | - | 0 | 0 | 2 | 10.0 | NE | - |
| Prior hospitalization and MRSA nasal carriage in the past 12 mo.^c^ |  |  |  |  |  |  |  |  |  |  |  |  |
| Not hospitalized in the past 12 mo. | 10 | 43.5 | 12 | 54.6 | 1.00 | - | 13 | 56.5 | 8 | 40.0 | 1.00 | - |
| Hospitalized and never screened positive for MRSA in the past 12 mo. | 8 | 34.8 | 6 | 27.3 | 0.47 | 0.10-2.34 | 9 | 39.1 | 5 | 25.0 | 0.72 | 0.12-4.38 |
| Hospitalized and screened positive for MRSA at least once in the past 12 mo. | 5 | 21.7 | 4 | 18.2 | 0.54 | 0.10-2.90 | 1 | 4.4 | 7 | 35.0 | 7.67 | 0.86-68.38 |
| Household members ^c^ |  |  |  |  |  |  |  |  |  |  |  |  |
| No household members | 4 | 17.4 | 3 | 13.6 | 1.00 | - | 4 | 17.4 | 3 | 15.0 | 1.00 |  |
| Household members did not use antibiotics in the past 4 weeks and not hospitalized in the past 12 mo. | 16 | 69.6 | 5 | 22.7 | 0.89 | 0.18-4.48 | 17 | 73.9 | 12 | 60.0 | 1.19 | 0.19-7.53 |
| Household members used antibiotics in the past 4 weeks and/or was hospitalized in the past 12 mo. | 3 | 13.0 | 14 | 63.6 | NE | - | 2 | 8.7 | 5 | 25.0 | 3.64 | 0.34-39.22 |

**Table S3.** Estimates of association of health care and community associated methicillin resistant *Staphylococcus aureus* (MRSA) nasal carriage with medical and household exposures from conditional logistic regression models

Abbreviation: months, mo.; odds ratio, OR; confidence interval, CI; non-estimable effect estimate, NE

^a^ Non-white or Hispanic includes non-Hispanic black, Hispanic/Latino, Asian, American Indian, or other race/ethnicities.

^b^The gym visitation/sports participation variable reflects the 2 weeks prior to the hospital admission.

^c^ Entered into the model as a 3-level categorical variable.
